# Ductoscopy Facilitates Surgical Decision-Making in Management of Patients with Pathological Nipple Discharge †

**DOI:** 10.3390/diagnostics16060856

**Published:** 2026-03-13

**Authors:** Vusal Aliyev, Zeliha Turkyılmaz, Enver Özkurt, Mehmet Durmus Kurt, Mustafa Tukenmez, Selman Emiroglu, Sibel Ozkan Gurdal, Beyza Ozcinar, Fatih Levent Balci, Omer Bender, Mahmut Muslumanoglu, Neslihan Cabioglu

**Affiliations:** 1 Department of General Surgery, Istanbul Faculty of Medicine, Istanbul University, 34452 Istanbul, Türkiye; dr.vusal@outlook.com (V.A.); turkyilmazz@yahoo.com (Z.T.); doctorenver@gmail.com (E.Ö.); drmehmetdurmuskurt@gmail.com (M.D.K.); mustafa.tukenmez@istanbul.edu.tr (M.T.); selman.emirikci.82@istanbul.edu.tr (S.E.); drasog@yahoo.com (S.O.G.); bozcinar@istanbul.edu.tr (B.O.); mahmutm@istanbul.edu.tr (M.M.); 2 Department of General Surgery, American Hospital, 34365 Istanbul, Türkiye; fatihleventbalci@gmail.com; 3 Department of General Surgery, Yeniyüzyıl University, 34010 Istanbul, Türkiye; omerbender@hotmail.com

**Keywords:** nipple discharge, mammary ductoscopy, intraductal papilloma, breast cancer, duct excision, papillomatosis

## Abstract

**Background/Objectives:** We investigated the feasibility of ductoscopy in diagnosis and management of patients presenting with clinically pathological nipple discharge (PND). **Methods:** Mammary ductoscopy was performed on 57 breasts with PND in 54 patients for diagnostic and therapeutic purposes. Ductoscopic abnormalities included ductal irregularities, presence of erythematous patches, or presence of intraductal papillomas, whereas duct ectasia or presence of dense fluid was considered a benign finding. **Results:** Age older than 40 and any pathology in ultrasound or ductoscopy were significantly associated with a decision of surgery. Surgical procedures included central duct excisions (*n* = 16) or specific duct excisions (*n* = 14) guided by either ultrasound (USG) or ductoscopy. Presence of an abnormal finding on ductoscopy was significantly associated with a specific lesion yield for PND in 18 patients, such as ductal carcinoma in situ with/without papillary cancer (*n* = 2, 11.1%), or intraductal papilloma/papillomatosis (*n* = 15, 83.3%) or periductal mastitis (*n* = 1, 5.6%) (specific lesion for PND; ductoscopic abnormality: 83.3% (15/18) vs. ductoscopic benign findings: 16.7% (2/12); *p* = 0.001) in patients undergoing surgical intervention. However, pathological findings in other imaging modalities including USG, magnetic resonance imaging (MRI) or mammogram were not associated with specific lesion yield for PND. The cancer detection rate in surgically excised lesions (*n* = 30) was 6.7% (*n* = 2). Overall, nipple discharge ceased in three patients who were conservatively observed after benign findings on ductoscopy at a median of 58 months (IQR, 39–77). **Conclusions:** The use of ductoscopy facilitated surgical decision-making in patients with PND, helping to distinguish patients requiring surgical excision from those suitable for conservative follow-up. In this cohort, ductoscopy findings were frequently associated with specific lesions, including mostly intraductal papilloma, explaining nipple discharge in patients selected for surgery.

## 1. Introduction

The third most common reason for admission to breast clinics, after complaints of breast pain and breast masses, is nipple discharge (ND). Approximately 2–6% of patients visit breast clinics with ND complaints [1,2]. Pathological nipple discharge (PND) is defined as spontaneous, unilateral, colored, or colorless discharge originating from a single duct, excluding cases related to pregnancy and breastfeeding [3,4].

PND often results from benign pathologies. The most common cause of PND is intraductal papilloma (IDP), with studies reporting the incidence of in situ and invasive cancer to be between 5% and 23% [4,5,6,7]. Another study found that the overall frequency of encountering malignant or high-risk pathologies in patients with PND was 15% [8]. This difference may be attributed to variations in patient selection across studies. While the probability of serous ND being caused by cancer is 7%, the frequency for bloody ND ranges between 5% and 28% [9]. Ductal carcinoma in situ (DCIS) is the most common breast cancer pathology encountered in patients with PND, rather than invasive breast cancer [6,10].

Different diagnostic methods, such as mammography (MMG), ultrasonography (USG), galactography, magnetic resonance imaging (MRI), and cytology, are used in the diagnosis of patients with ND [4,11]. However, these tools often fail to identify intraductal lesions due to high false negative and positive rates and are unable to distinguish between benign and malignant intraductal lesions [4,6,11,12,13,14]. Conversely, breast milk ducts can be directly visualized using an endoscopy device (Ductoscope, Mammoscope), allowing for the evaluation of possible epithelial abnormalities and lesions, as well as the determination of their localization [1,15,16,17]. Additionally, in appropriate cases, IDP can be excised endoscopically without the need for additional surgery [1,15,16].

Mammary ductoscopy provides direct visualization of the ductal epithelium, which is the source of most papillary and malignant lesions. Ductoscopy has been shown to increase the detection of intraductal lesions in patients with PND, complementing other imaging techniques such as galactography, MRI, MMG, and USG. Therefore, we investigated the feasibility of ductoscopy in the diagnosis and management of patients presenting with clinical PND.

## 2. Methods

### 2.1. Patient Selection

Mammary ductoscopy was performed on 57 breasts with PND in 54 consecutive patients for diagnostic and therapeutic purposes in our Breast Clinic (Department of General Surgery, İstanbul University, İstanbul Faculty of Medicine) between 2008 and 2014. Three patients presented with bilateral ND. Local ethics committee approval was obtained for this study (2025/1253). All patients were informed about the procedure and their consent was obtained. This study follows the STROBE statement for cohort studies [18].

A denominator-explicit participant flow table was constructed to clarify the testing, verification structure, and denominators underlying the reported outcomes in accordance with STARD principles (Table 1).

Patients who presented to our clinic with PND were evaluated for the necessity of ductoscopy. This study included patients with spontaneous or induced ND (serous, bloody, serohemorrhagic, brown, green, yellow, milky) and those with ND and non-palpable breast masses. Patients using medication that could increase nipple secretion and patients with a breast mass > 1 cm on physical examination were excluded from this study. The findings from imaging studies and the results of cytological examinations of nipple aspirate fluid were recorded. The clinical characteristics of ND were evaluated along with other imaging and cytological tests to classify the discharge as clinically “benign” or “pathological”.

**Figure 1:**
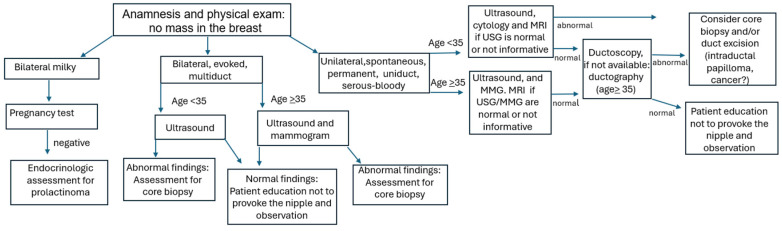
Algorithm illustrating the diagnostic and management pathway for patients presenting with pathological nipple discharge, including imaging evaluation, ductoscopy findings, and subsequent decisions regarding surgical intervention or conservative follow-up.

Ductoscopy was performed on patients with clinically diagnosed PND where no obvious lesion causing the discharge was detected on USG and MMG, as well as on patients with a suspicious intraductal lesion on USG or other imaging for which a definitive diagnosis could not be made. Patients classified in the clinically benign group were followed conservatively without surgical intervention, and were not included in this study. These patients were observed with clinical examinations and USG every 6 months, along with annual MMG, until the ND complaint ceased or was judged to be due to a benign etiology according to our clinical algorithm (Figure 1).

### 2.2. Device and Technique

A LaDuScope-T flex (Polydiagnost GmBH, Pfaffenhofen, Germany) with 60× magnification, a diameter of 0.9 mm, a working channel of 0.2 mm, and 6000 pixels was used for ductoscopy. The nipple–areola complex was cleaned with a povidone–iodine solution, and all procedures were performed in the operating room under local anesthesia. After appropriate dilation of the nipple using dilators, the endoscope was directed into the ductal space. Saline solution was injected to expand the duct and obtain a clear image of the intraductal space. During the procedure, ductal lavage was performed in 19 cases (35%) under ductoscopy guidance. Ductoscopic abnormalities included ductal irregularities with erythematous patches or intraductal papillomas, whereas duct ectasia or the presence of dense fluid was considered a benign finding.

### 2.3. Radiological Methods

USG was performed on all patients who presented with ND. Bilateral MMG (*n* = 45) was conducted for patients over the age of 35. Some patients with dense MMG or no pathology detected on MMG or USG also underwent contrast-enhanced MRI (*n* = 18). Mammographic pathological findings included microcalcifications, abnormal structural distortions, asymmetric density increases, and excessive dilation/ductal ectasia of the breast ducts, particularly when BIRADS IV/V was present on the side with PND in MMG. Suspicious retroareolar intraductal mass or ductal dilation observed on ultrasound was considered a pathological finding. Ultrasonographic abnormalities included the presence of a retroareolar intraductal mass, such as papilloma or papillomatosis, suspicious solid malignant lesions (BIRADS IV&V), or ductal dilation. Retroareolar pathological contrast enhancement or suspicion of an IDP was considered a pathological MRI finding.

Ductography was performed in three patients in whom no pathology was detected on MMG or USG to investigate PND. After cannulating the duct with pathological discharge, craniocaudal and mediolateral mammograms were conducted using 0.5–1.5 cc of water-soluble contrast material (Cysto-Conray 60%; Mallinckrodt Inc., St Louis, MO, USA) administered through the ductography catheter. Pathological findings assessed included filling defects, ductal ectasia-dilatation, ductal constriction, ductal irregularities, and multiple filling defects.

### 2.4. Cytological Examinations

ND cytology was performed in 30 cases (53%). Similarly, ductal lavage was performed in 19 out of 55 successful ductoscopies (34.6%). Pathological findings in ND and ductal lavage cytology included cells containing papillary, atypical, suspicious, or malignant features. Benign findings were characterized by the presence of acellular ductal cells, foam cells, inflammatory cells, and blood elements.

### 2.5. Statistical Analysis

The clinical characteristics of patients along with imaging, ductoscopic and pathological findings were analyzed retrospectively. Analyses were performed blinded when interpreting imaging and ductoscopic findings. Statistical analyses of this study were performed using the SPSS 17 (Statistical Package for Social Sciences; SPSS, Inc., Chicago, IL, USA) statistical software program. *p* < 0.05 was considered statistically significant. Fisher’s test was used for categorical analyses, and *p*-values in two-way analyses were considered for the statistical significance. Analyses were primarily performed at the patient level. Because three patients underwent bilateral ductoscopy, breast-level findings are presented descriptively. Due to the limited sample size, clustering effects related to bilateral observations could not be formally adjusted, which could represent a potential limitation.

## 3. Results

Fifty-four consecutive patients with complaints of PND were included in this study. Among them, 52 (96.3%) were women, and 2 were male patients with spontaneous bloody ND. The median age was 46 years (range 12–76). The clinic-demographic characteristics of the patients are summarized in Table 2. Thirty-four patients (65%) were in the premenopausal period. Two patients had a previous history of contralateral breast cancer, who were diagnosed with intraductal papilloma. All discharges were single-channel, 93% were spontaneous, 94% were unilateral, and 84% were colored, bloody, serous, and serohemorrhagic. The majority of cases (48/57, 84%) presented with spontaneous uniduct bloody or serous discharge. The average duration of ND complaints was 4 (1–120) months. Physical examination findings other than ND were not evident in 95% of the cases.

Surgical or conservative management decisions were made based on combined clinical, imaging, and ductoscopic findings. Of 54 patients evaluated for PND, ductoscopy was attempted in 57 breasts. Surgical intervention was performed in 30 breasts based on combined clinical, imaging, and ductoscopic findings, whereas 27 breasts were managed conservatively with follow-up after benign findings.

Radiological findings are summarized in Table 3. USG, MMG, MRI, and ductography were performed in 98.1% (53/54), 81.5% (44/54), 33.3% (18/54), and 5.6% (3/54) of the patients, respectively. Among the patients who underwent ductoscopic procedures, imaging findings were normal in MMG, USG, MRI, or ductography in 63.6% (28/44), 21% (11/53), 38.9% (7/18), and 33.3% (1/3) of cases, respectively. Importantly, imaging findings associated with a papilloma were detected in MMG, USG, MRI, or ductography in 6.8% (3/44), 22.5% (12/53), 38.9% (7/18), and 66.6% (2/3) of patients, respectively.

Ductoscopy was attempted in a total of 57 breasts from 54 patients, with 3 patients undergoing bilateral procedures. The procedure could not be performed in two breasts (3.5%) due to nipple duct stenosis or rupture. Ductoscopic results are detailed in Table 3. Normal findings were observed in 12 breasts (21.1%), while intraductal debris was detected in 11 cases (19.3%) and ductal irregularities in 9 cases (15.8%). Saline irrigation was used for cases with intraductal debris. Intraductal papilloma (IDP) was the most common finding, identified in 15 procedures (26.3%). Malignancy was identified in two cases (6.7%). Histopathology revealed one case of low-grade ductal carcinoma in situ (DCIS) associated with papillary carcinoma and one case of DCIS with microinvasion, corresponding to ductoscopic findings of ductal irregularities with erythematous patches. However, one patient with ductoscopic findings suspicious for invasive malignancy was found to have intraductal papilloma (IDP) on the final pathology, representing a discordant result (Table 4). Surgical intervention was primarily performed for cases suspicious of IDP or malignancy based on ductoscopic findings. Overall, surgical intervention was conducted in 30 breasts out of 57 procedures (52.6%), based on clinical symptoms such as ND complaints, physical examination findings including ND characteristics, and other imaging results.

Factors predicting the decision for surgery in patients with PND are summarized in Table 5. Age older than 40 years (surgery positive; >40 age: 90% vs. ≤40 age: 63%, *p* = 0.025), abnormalities detected in USG (surgery positive; USG abnormality: 45% vs. other: 8%, *p* = 0.005), MRI (surgery positive; MRI abnormality: 60% vs. other: 12.5%, *p* = 0.066), and ductoscopy (ductoscopic abnormality: 70% vs. benign ductoscopic finding: 32%, *p* = 0.007) were identified as significant factors associated with a decision for surgery for the diagnosis and treatment of PND. Overall, abnormal cytology findings were detected in two cases with unilateral disease in the surgery group (13.3%).

Preoperative fine-needle aspiration (FNA) or core biopsies were conducted for patients with suspicious lesions detected during ductoscopy. In one case, a papillary lesion identified by FNA was subsequently marked with a wire, leading to surgical excision. Histopathological examination confirmed intraductal papilloma (IDP). Another patient with suspected malignancy underwent surgical excision due to insufficient biopsy material. During the operation, segmental mastectomy and sentinel lymph node biopsy (SLNB) were performed. A biopsy of adenosis in one patient resulted in a decision for conservative management, with follow-up scheduled every 6 months for the first 2 years, followed by annual monitoring without surgical intervention.

Surgical procedures included central duct excisions (*n* = 16) or specific duct excisions (*n* = 14), guided by either ultrasound or ductoscopy (Table 6). Following central duct excision, one patient diagnosed with male breast cancer in a frozen section underwent mastectomy with sentinel lymph node biopsy. Additionally, eight patients underwent intraoperative ductoscopy, guiding excision under direct visualization (Figure 1).

**Figure 2:**
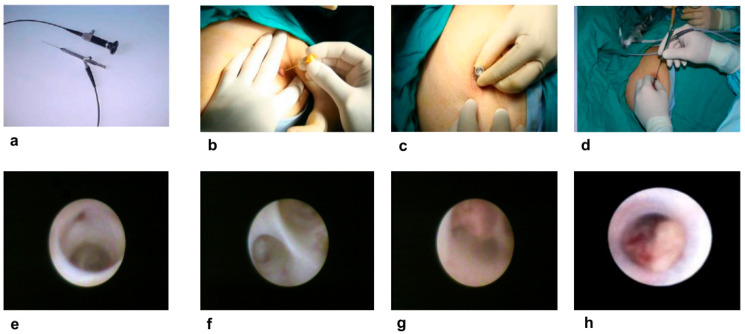
(**a**) LaDuScope-T flex (Polydiagnost GmBH, Pfaffenhofen, Germany); (**b**) obturator for Solex-nipple expander before duct cannulation that alleviates the insertion of the Solex-nipple lumen expander (Polydiagnost GmBH, Pfaffenhofen, Germany); (**c**) duct cannulation with a Solex-nipple lumen expander that alleviates the insertion of the ductoscope (Polydiagnost GmBH, Pfaffenhofen, Germany); (**d**) ductoscopy-guided duct excision; (**e**) normal ducts in the nipple orifice; (**f**) bifurcation of the normal ducts; (**g**) intraductal papilloma after the first bifurcation of the ducts; (**h**) an obstructive intraductal papilloma at the orifice of the nipple.

**Figure 3:**
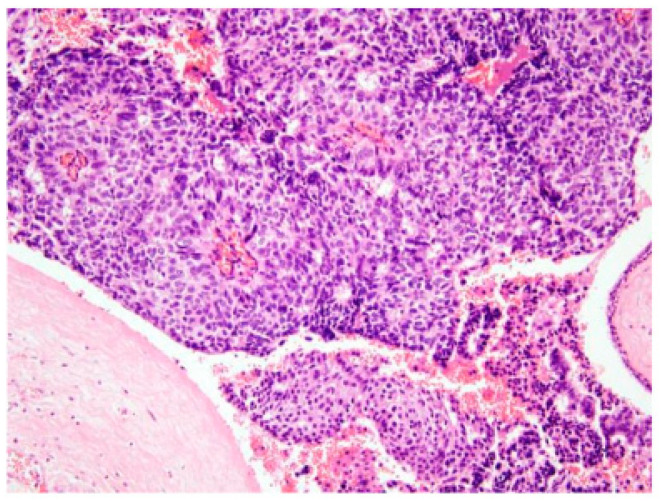
Papillary cancer and DCIS; H&E 200×.

The most common excised lesion was intraductal papilloma (Figure 2f–h). Histopathological findings are detailed in Table 7. Overall, a specific lesion associated with PND was identified in 60% of cases (*n* = 18) of DCIS with/without papillary cancer (*n* = 2, 11.1%, Figure 3), or intraductal papilloma/papillomatosis with/without atypical ductal hyperplasia (*n* = 15, 83.3%) or periductal mastitis (*n* = 1, 5.6%). Therefore, the cancer detection rate in surgically excised lesions (*n* = 30) was 6.7% (*n* = 2).

Furthermore, the presence of an abnormal finding on ductoscopy significantly correlated with underlying specific pathological lesions associated with PND (specific lesion for PND (+); ductoscopic abnormality: 83.3% (15/18) vs. ductoscopic benign findings: 16.7% (2/12); *p* = 0.001) in patients undergoing surgical intervention. However, no significant associations could be found between the specific lesion yield and pathologies in other imaging methods, including ultrasound, mammogram and MRI. The specific lesion yields for PND were 50% (9/18) (vs. 36.4%, 4/11, *p* = 0.702) in pathological findings of USG, 83.3% (5/6) (vs. 25%, 1/4, *p* = 0.190) in pathological findings of MRI, and 33.3% (5/15) (vs. 36.4%, 4/11, *p* = 0.999) in pathological findings of MMG, respectively.

Moreover, there was no significant difference observed between patients who underwent a localization technique and those who did not (with localization: 9/16 (56.3%) vs. without localization: 9/14 (64%), *p* = 0.722) in obtaining specific lesions associated with PND.

In this cohort, abnormal ductoscopic findings were frequently associated with histopathological lesions, explaining pathological nipple discharge among surgically treated patients. Because histological verification was mainly available in patients undergoing surgery, these findings should be interpreted as descriptive outcomes rather than comparative diagnostic performance estimates.

Nipple discharge ceased in three patients who were conservatively observed after benign findings on ductoscopy at a median of 58 months (IQR, 39–77). However, all patients who were conservatively managed after benign ductoscopic findings did not experience any PND. Of those (*n* = 43) with a longer follow-up more than 5 years, two patients developed breast cancer (4.6%) at a median of 155 months (IQR, 135–180).

## 4. Discussion

Patients with PND have long posed a diagnostic and therapeutic challenge to surgeons. In diagnosing PND, indirect imaging methods such as MMG, USG, MRI, ductography, and discharge smear are still occasionally used in most centers [19,20]. A study reported that cancer was detected in only 2% of patients with suspicious ND after three years of follow-up when no pathology was found in both MMG and USG [19].

Although this suggests that MMG and USG may be sufficient to detect pathology in ND in most cases, data from many studies do not support such a high diagnostic value for both USG and MMG. The sensitivity of MMG in detecting abnormalities in patients with PND is quite low. The underlying reason for this situation is that the lesions are often small, intraductal, and free of calcification. In a study where 96% of 273 patients with ND underwent MMG, the sensitivity of MMG was found to be 15%, while the specificity was 98% [6].

There is no consensus on the value of USG in diagnosing PND. The sensitivity and specificity of USG vary widely, ranging at 43–82% and 31–75%, respectively [4,6,21,22]. In a study by Bahl et al., the sensitivity and specificity of USG in detecting DCIS and invasive adenocarcinoma were reported as 56% and 75%, respectively [6]. In our study, the rates of pathological findings in MMG and USG to decide on surgery in diagnosing PND were 35% and 45%, respectively. Intraductal debris was detected ductoscopically in 11 patients whose USG examination revealed benign findings such as a dense content and ductal ectasia. Only two of these patients underwent surgical resection, and IDP was detected in one of them. These lesions detected on USG are also seen as IDP or filling defects on galactography, leading to potential false findings and an indication for surgical excision.

Many studies have shown cytology to be a low-level diagnostic technique when used alongside standard MMG/USG for PND [4,23]. The sensitivity of PND cytological examinations is reported to be around 15% [24]. Similarly, any pathological finding of cytology was detected in 13% of cases in the surgery group. Various studies suggest using fluorescence in situ hybridization (FISH) [25] or the DNA index and S-phase fraction [26], or performing chromosomal anomaly tests such as methylation-specific PCR [27] to increase the sensitivity and the specificity of ductal cytology.

Many previous studies have concluded that radiological examinations, including MRI and ductography, are not sensitive in diagnosing patients with PND [8,28]. The added value of breast MRI in demonstrating malignancy in patients with unilateral bloody ND, when no signs of malignancy are found on MMG or USG, is extremely limited [29]. However, some authors report that breast MRI shows significantly higher overall sensitivity values compared to MMG and USG, and they recommend MRI in the presence of suspected PND when no findings are detected in MMG and USG [11,30]. Overall, the pooled sensitivity for MRI is reported to be 92% and the specificity 76% [20]. In a study of patients with no abnormalities detected in conventional imaging, MRI and ductography were compared, with MRI showing a sensitivity of 95.7% and ductography of 85.7% [31]. The sensitivity of galactography in detecting lesions varies between 49% and 100%, while the specificity varies between 12% and 100% [4,30]. A meta-analysis reported that the pooled sensitivity and specificity were 69% and 39%, respectively [30]. MRI offers practical advantages such as improved patient compliance, ease of application, and visualization of both breasts and the axilla [30]. In our study, the frequency of pathological findings on MRI was 60% in the surgery group versus 12.5% in the follow-up patient group.

In a ductoscopy series first reported in 1991 by Okazaki et al., direct visualization of the breast ducts was made possible by ductoscopy, and it has since become increasingly widely used [32]. While the inner surface of a normal duct is shiny and smooth, intraductal solid nodules detected on the duct wall appear as yellow papillomas or red if bleeding areas are present. If cancer has developed in the duct wall, it appears as white, slightly raised lesions. Endoscopic evaluation of intraductal lesions can provide an early diagnosis of cancer and significantly contribute to the evaluation of nipple involvement in intraductal carcinoma [32]. Since ductoscopy can enlarge breast tissue up to 60 times its actual size, it allows for the detection of much smaller lesions compared to those detected by MMG and MRI [33].

Fisher et al. found that atypia and malignancy were diagnosed in 7% of patients with PND, who had no suspicion of malignancy in routine imaging findings, by ductoscopy [34]. Dietz et al. reported that all PND patients with cancer presented with bloody or brown ND, whereas in the study conducted by Fisher et al., most of the patients had bloody ND, while non-bloody ND was detected in 33% [34,35]. In our series, 40% of the discharges were bloody and 31.6% were serous.

Age is an important factor in increasing the risk of breast cancer in patients with ND. Similarly, a study conducted at the M.D. Anderson Cancer Center found that being under 40 years of age was associated with benign ND [4]. Some studies report that a menopausal status may be an independent variable that increases the risk of malignancy, but the risk cannot be ignored in patients with pre-menopausal PND [34]. In the present study, ductoscopy served as a crucial diagnostic tool in determining the need for surgical intervention. Our findings suggest that an age younger than 40 and detecting benign findings on ductoscopy or USG should be considered criteria for conservative follow-up without surgery.

Although major duct excision often provides a definitive result in treatment, it also carries risks such as loss of nipple sensation and possible areolar necrosis of the nipple [1]. The advantage of ductoscopy is that it allows direct visualization of intraductal lesions and enables biopsies to be performed when necessary. Therefore, selective surgery for a duct with a suspected pathology detected by ductoscopy is a safer option.

Ductoscopy may help reduce the number of unnecessary surgical canal excisions and may provide advantages such as reducing the extent of surgical resection [24]. Although ductoscopy can easily reach the main central ducts that drain most of the breast volume, it may be insufficient to examine the terminal duct-lobular unit where malignant lesions originate [24]. In a study by Dooley, in which routine intraoperative ductoscopy was used during lumpectomy, 74.6% of cases could be successfully dilated and fluoroscopy performed, reducing the incidence of positive margins from 23.5% to 5.0% [36]. Histopathological diagnosis is still essential to exclude malignancy in patients with PND and positive ductoscopy findings. However, studies show that this diagnosis can be made with endoscopic biopsy [14]. In cases where endoscopic tissue diagnosis is not possible, surgery is still inevitable if a ductal lesion is present [14].

In our cohort, ductoscopy frequently identified intraductal abnormalities in patients selected for surgery after conventional imaging yielded inconclusive results. These findings support a complementary role of ductoscopy in clinical decision-making rather than demonstrating comparative diagnostic superiority. Previous studies have reported variable detection rates for different diagnostic approaches in patients with PND [37]. As expected, our study also found a higher rate of cases with specific lesions associated with PND (PND-specific lesion-positive) in those with pathological ductoscopic findings necessitating surgical duct excision (83.3% versus ductoscopic benign findings; 16.7%, *p* = 0.001). A study from the MD Anderson Cancer Center [4] previously reported that among patients undergoing surgery for PND, those who underwent ductography-guided operations (*n* = 42, 50%) or any surgical procedure including localization studies (*n* = 66, 78.6%) were significantly more likely to have specific lesions associated with PND compared to those undergoing central duct excision (*p* = 0.045 and *p* = 0.033, respectively). However, in the present study, no significant difference was found in terms of obtaining specific lesions for PND between patients who underwent surgery including localization procedures such as wire localization or ductoscopic guidance and those who did not (localization positive, 56.3% versus localization study negative, 64%). Preoperative ductoscopy was applied to all cases in our study. Therefore, preoperative or perioperative localization of lesions might have been necessary only for those with peripheral lesions or in young premenopausal patients to avoid extensive surgical excisions such as central duct excision. Consequently, additional ductoscopic marking in lesions near the nipple was unnecessary. Using this approach, our series found a high rate (57%) of specific lesions (intraductal papilloma and malignancy) for PND, consistent with the literature [4].

Of note, in the current study, surgical intervention was omitted in patients with normal or benign ductoscopic and/or USG findings, as illustrated in Figure 1. These patients were closely monitored with USG every 6 months without undergoing surgery, along with annual MMG over an average of 5 years, and ND ceased without recurrence. A cohort study of patients with PND who underwent ductoscopy with normal findings showed no recurrence of PND or cancer during a 4-year follow-up period [38].

A recent study suggested that if US and MMG yield negative results in the presence of PND, MRI should be the preferred subsequent examination rather than galactography. It reported that MRI’s high sensitivity and high negative predictive value make it sufficient for selecting patients suitable for surgery and observation. The same study emphasized that ductoscopy, with its high sensitivity and also negative predictive value reaching 98–100%, eliminates the need for surgery in patients with normal findings on ductoscopy [39].

The limitations of the present study include its retrospective design and potential selection bias of the cohort since the criteria for referral to ductoscopy inherently selected a higher-risk subgroup. Although consecutive patients are included, selection bias of patients due to our clinic as a referral center for ductoscopy could limit generalizability and inflate diagnostic yield estimates. Because histopathological confirmation was primarily available in patients undergoing surgery, diagnostic performance measures may be influenced by verification and work-up bias. Therefore, findings should be interpreted as reflecting management impact rather than general diagnostic accuracy.

Despite its advantages, the need for experience in performing ductoscopy and the lack of knowledge in interpreting ductoscopic images hinder its widespread use. Therefore, increasing the number of studies and publications related to ductoscopy and image interpretation will raise interest in this technique. In the future, technological advancements may lead to thinner endoscopes capable of accessing more advanced areas of the ducts, thereby enhancing the effectiveness of the technique in detecting lesions. In recent years, a small number of studies have reported that digital breast tomosynthesis shows high sensitivity and high specificity. Furthermore, contrast-enhanced mammography is reported to show higher sensitivity and specificity than combined MMG and US in patients with bloody nipple discharge; however, more studies are needed to strengthen its place in daily practice [12]. Recent high-quality meta-analyses and comparative studies have further clarified the role of advanced imaging modalities in the evaluation of pathological nipple discharge. Evidence suggests that breast MRI demonstrates an improved diagnostic performance compared with conventional galactography in selected patients and may enhance clinical decision-making when standard imaging is inconclusive [30,40]. In addition, contemporary management strategies support selective surgical excision guided by structured diagnostic algorithms to reduce unnecessary duct excisions while maintaining oncological safety [41,42]. These data collectively support a tailored, evidence-based approach rather than routine surgical intervention in all patients with PND.

## 5. Conclusions

In this retrospective cohort of patients undergoing ductoscopy within a defined clinical pathway for pathological nipple discharge, abnormal ductoscopic findings were frequently associated with histologically confirmed intraductal lesions in the surgically treated subgroup. Ductoscopy contributed to surgical decision-making and lesion localization within this selected population.

Because histological confirmation was predominantly available in operated patients and testing was not uniform across the cohort, the comparative diagnostic accuracy between modalities cannot be inferred. The findings should therefore be interpreted as cohort-conditional observations regarding management and the lesion yield rather than as estimates of the relative test performance.

## Figures and Tables

**Table 1:** Participant flow.

Characteristic	Number
Consecutive patients with pathological nipple discharge	54
Breasts with attempted ductoscopy	57
Successful ductoscopy	55
Unsuccessful ductoscopy due to the cannulation failure or duct rupture	2
Surgical intervention with histopathological confirmation	30
Follow-up without surgery	27
Median follow-up duration	58 months (IQR, 39–77).
Cancer detected during follow-up	2

**Table 2:** Clinico-demographic characteristics of the cohort with nipple discharge who underwent attempted ductoscopy.

Characteristic	*N* = 54
Median age (range, min–max)	46 (12–76)
Median nipple discharge duration, months	4 (1–120)
Female, *n* (%)	52 (96.3%)
Male, *n* (%)	2 (3.7%)
Menopausal status: Premenopausal Postmenopausal	34 (63%) 18 (33.3%)
History of breast cancer: Yes No	2 (3.7%) 52 (96.3%)
History of previous birth: Yes No	46 (85.2%) 6 (11.1%)
Median number of births:	3 (1–11)
Breastfeeding: (*n* = 46) Yes No	39 (84.8%) 7 (15.2%)
Nipple discharge side: Bilateral Unilateral	3 (5.6%) 51 (94.4%)
Nipple discharge color: Bloody Serous Other	23 (42.6%) 18 (33.3%) 13 (24.1%)
Characteristic of the nipple discharge: Spontaneous Evoked (induced)	53 (93%) 4 (7%)

**Table 3:** Imaging findings in the cohort with nipple discharge who underwent attempted ductoscopy.

	*N* (%)
**Mammography (*****n***** = 44)** Normal Fibrocystic change Ductal prominence Duct wall thickening Retroareolar tubular opacity Intraductal papilloma Asymmetric density	28 (63.6) 3 (6.8) 6 (13.6) 1 (2.3) 1 (2.3) 3 (6.8) 2 (4.5)
**Ultrasonography (*****n***** = 53)** Normal Cyst Ductal ectasia Intense content and ductal ectasia Ductal ectasia and solid component Intraductal papilloma and papillomatosis	11 (20.8) 8 (15.1) 14 (26.4) 5 (9.4) 3 (5.7) 12 (22.6)
**Ductography (*****n***** = 3)** Normal Tubular contrast enhancement Filling defect	1 (33.3) 1 (33.3) 1 (33.3)
**Magnetic resonance imaging (*****n***** = 18)** Normal Ductal ectasia Intraductal papilloma Diffusion restriction	7 (38.9) 3 (16.7) 7 (38.9) 1 (5.6)

**Table 4:** Ductoscopic findings (*n* = 57 breasts) and corresponding surgical pathology (*n* = 30 breasts) in the cohort with nipple discharge who underwent attempted ductoscopy.

Ductoscopic Findings	*N* = 57 (%)	Pathology Findings After Surgery (*n* = 30)
Normal	12 (21.1)	Fibrocystic changes (*n* = 1), ductal ectasia (*n* = 3), no surgery (*n* = 8)
Debris	11 (19.3)	Intraductal papilloma (*n* = 1), ductal ectasia (*n* = 2), periductal mastitis (*n* = 1), no surgery (*n* = 7)
Ductal irregularity (with duct ectasia, *n* = 2, with duct narrowing, *n* = 2)	9 (15.8%)	Intraductal papilloma (*n* = 1), columnar changes (*n* = 1), ductal ectasia (*n* = 1), no surgery (*n* = 6)
Intraductal papilloma	15 (26.3)	Intraductal papilloma (*n* = 10), intraductal papilloma with atypical hyperplasia (*n* = 1), papillomatosis (*n* = 1), ductal ectasia (*n* = 2), adenosis (*n* = 1)
Patchy erythematous duct irregularities	2 (3.5)	Ductal carcinoma in situ (*n* = 2)
Malignancy	1 (1.8)	Papillomatosis (*n* = 1)
Ductal narrowing (with erythema, *n* = 1)	2 (3.5)	Ductal ectasia (*n* = 1), no surgery (*n* = 1)
Ductal ectasia	2 (3.5)	No surgery (*n* = 2)
Periductal mastitis	1 (1.8)	No surgery (*n* = 1)
Rupture	1 (1.8)	No surgery (*n* = 1)
Could not be cannulated	1 (1.8)	No surgery (*n* = 1)

**Table 5:** Factors predicting the decision for surgery in patients presenting with nipple discharge (54 patients with 57 breast ductoscopies).

	Observation, *n* = 27 (%)	Surgery, *n* = 30 (%)	*p*
**Age**			0.025
≤40	10/27 (37)	3/30 (10)	
>40	17/27 (63)	27/30 (90)	
**Discharge color**			0.280
Bloody/serous	21/27 (78)	27/30 (90)	
Others (green, yellow, milk, etc.)	6/27 (22)	3/30 (10)	
**Physical examination**			0.239
Mass (−)	27/27 (100)	27/30 (90)	
Mass (+)	0/27 (0)	3/30 (10)	
**Ductoscopy (** ***n*** = 55 *)			0.007
Pathological (ductal irregularity, intraductal papilloma, etc.)	8/25 (32)	21/30 (70)	
Benign (normal, heavy content, ductal ectasia)	17/25 (68)	9/30 (30)	
**Ultrasonography**			0.005
Pathological (intraductal papilloma, etc.)	2/24 (8)	13/29 (45)	
Benign (cyst, ductal ectasia, etc.)	22/24 (92)	16/29 (55)	
**Magnetic resonance imaging**			0.066
Pathologic	1/8 (12.5)	6/10 (60)	
Benign	7/8 (87.5)	4/10 (40)	
**Mammography**			0.507
Pathologic	4/18 (22)	9/26 (35)	
Benign	14/18 (78)	17/26 (65)	
**Cytology (** ***n*** ** = 30)**			0.999
Pathologic	2/15 (13)	2/15 (13)	
Benign	13/15 (87)	13/15 (87)	

* Ductoscopy could not be completed in two cases due to rupture or unsuccessful cannulation of the nipple duct.

**Table 6:** Surgical intervention in patients with pathological nipple discharge.

Surgical Type	*N* = 30 (%)
Central duct excision *	15 (50)
Duct excision with wire localization *	11 (37)
Mass excision with wire localization *	3 (10)
Central duct excision (DCIS and microinvasion) followed by mastectomy and sentinel lymph node biopsy	1 (3)

* with perioperative ductoscopy (*n* = 8, 26.7%).

**Table 7:** Pathological findings after surgery (*n* = 30).

Pathological Findings	*n* (%)
**Specific lesion for pathological nipple discharge:**	18 (60)
Intraductal papilloma and papillomatosis	14
Intraductal papilloma and atypical ductal hyperplasia	1
Papillary carcinoma and DCIS (low grade)	1
DCIS and microinvasion	1
Periductal mastitis	1
**Benign lesions not specific to pathological nipple discharge:**	12 (40)
Ductal ectasia	9
Periductal mastitis with columnar changes and ductal ectasia	1
Fibrocystic changes	1
Adenosis	1

## Data Availability

The original contributions presented in this study are included in the article. Further inquiries can be directed to the corresponding author.
